# Prevalence of and factors affecting malocclusion in primary dentition among children in Xi’an, China

**DOI:** 10.1186/s12903-016-0285-x

**Published:** 2016-09-02

**Authors:** Zhifei Zhou, Fen Liu, Shuning Shen, Linjuan Shang, Lei Shang, Xiaojing Wang

**Affiliations:** 1 State Key Laboratory of Military Stomatology, National Clinical Research Center for Oral Diseases, Department of Pediatric Dentistry, School of Stomatology, Fourth Military Medical University, Xi’an, People’s Republic of China; 2 Department of Oral Medicine, Northwest Women’s and Children’s Hospital, Xi’an, People’s Republic of China; 3 Department of Oral Medicine, PLA 261 Hospital, Beijing, People’s Republic of China; 4 Department of Pediatric Dentistry, Foshan Stomatological Hospital, Foshan, People’s Republic of China; 5 Department of Health Statistics, School of Public Health, Fourth Military Medical University, Xi’an, People’s Republic of China

**Keywords:** Cross-sectional study, Epidemiology, Malocclusion, Prevalence, Primary dentition

## Abstract

**Background:**

This study aimed to investigate the prevalence and associated factors of malocclusion among children with primary dentition in Xi’an, China.

**Methods:**

A total of 2,974 subjects were selected from local schools in Xi’an city using a stratified cluster sampling method from January to September 2015. After screening samples according to the inclusion criteria, the final sample size comprised 2,235 pre-school children, with a mean age of 4.82 (SD, 1.76; range, 2.63–6.12) years. Malocclusion traits were assessed by trained clinicians followed by the evaluation of associated factors through clinical examination and a precisely designed questionnaire including data regarding gender, birth place, parental education level, monthly familial income, parental attitude toward the problem of malocclusion, feeding methods of the children, feeding postures, pacifier use, and delivery methods. Data were analyzed using the Chi-square test and logistic regression analysis.

**Results:**

The most common type of malocclusion was increased overjet (34.99 %) in the sagittal direction, deep overbite (37.58 %), and midline deviation (25.32 %) in the vertical and transverse directions, respectively. The prevalence of posterior crossbite, anterior crossbite, and anterior open bite was 7.56, 6.80 and 6.98 %, respectively. The prevalence of the anterior edge-to-edge occlusion was the lowest (2.46 %). The variables associated with malocclusion (*P* < 0.05) were birth place (odds ratio [OR] = 1.741 with 95 % CI of 1.384–2.162), insufficient abrasion of primary canines (OR = 1.465; 95 % CI of 1.153–1.894), caries in primary teeth (OR = 2.045; 95 % CI of 1.665–2.539), tongue thrusting (OR = 2.833; 95 % CI of 1.640–3.649), mandibular prognathism (OR = 2.621; 95 % CI of 1.574–3.689), and finger sucking (OR = 1.573 with 95 % CI of 1.098–2.014). The feeding methods (OR = 3.614 with 95 % CI of 3.087–4.596) along with the method of delivery (OR = 1.847 with 95 % CI of 1.323–2.451) have been observed to play an important role in the morbidity of malocclusion (*P* < 0.05).

**Conclusions:**

The prevalence of malocclusion among pre-school children in Xi’an is higher compared to that in other geographical parts of China. Therefore, early attention to the development of occlusion and necessary interventions toward the associated factors are important to reduce its prevalence and further adverse effects.

## Background

Malocclusion is a condition characterized by abnormal relationships among the teeth or dentitions. It is one of the most common problems affecting the human oral cavity along with caries, gingivitis, and dental fluorosis [1]. It leads to symptoms such as deficient chewing, speech articulation, undesirable development of the jaw bones [2], etc. The prevalence of malocclusion is usually high among adolescents with permanent or mixed dentition. Kaur et al. reported a high prevalence of malocclusion (87.79 %) among Indian adolescents aged 13–17 years [3]. The prevalence of normal occlusion among Nigerian children (aged 13–20 years) was only 11.8 % in 2014 [4]. The total prevalence of Class I, II, and III types of malocclusion among Iranian children aged 11–14 years old was 77.1 % [5].

Some cohort studies have already indicated that malocclusion in primary dentition is the determinant of malocclusion in permanent dentition [6–8]. Moreover, it would also increase the potential needs of orthodontic treatment in permanent dentition [9]. In addition, malocclusion may lead to low self-esteem in later years, as children with primary dentition undergo a period during which they establish self-identity and early personality [10]. The prevalence of malocclusion in primary dentition is more than 50 % worldwide [11, 12], and whether malocclusion during this period needs early management is debatable [9, 13]. However, scholars have confirmed that the severity of malocclusion in primary dentition would affect permanent dentition; thus, rather than early management, it is more important to identifying the influencing factors of malocclusion in this period and implement some preventive strategies [14, 15], in order to decrease the severity and lower the prevalence of malocclusion in future.

Therefore, proper and timely evaluation of the factors associated with malocclusions in the primary dentition could help in the prevention and better management of occlusion-related problems later in life [16]. Malocclusion is generally considered as a multifactorial condition that is linked to developmental factors (such as nasal obstruction impairing the nasal breathing), habits (such as thumb sucking), genetics, hereditary factors, and ethnicity [13, 17]. To date, very limited information is available regarding the prevalence of malocclusion among children with primary dentition in Chinese populations. Moreover, because of the different criteria and investigated indices, previous studies have reported different results among various ethnic groups [18, 19].

Currently, there is lack of information regarding the prevalence of malocclusion among the residents of Xi’an city. This epidemiological study aimed to investigate the prevalence and associated factors of malocclusion in primary dentition among children in Xi’an.

## Methods

### Sample selection

Xi’an is a main regional city in Northwest China, with an area of 10,108 square kilometers and a diverse population of more than eight million. A cross-sectional study was conducted in Xi’an city from January to September 2015. A total of 24 kindergarten schools registered with the pre-school education administration institute in the northern, central, and western districts of Xi’an city were randomly selected. From each kindergarten, two classes in each grade were randomly selected. All children of the selected classes (*n* = 2,974) were included in this study. Children with fully erupted primary dentition and without any permanent teeth including the first molars or permanent incisors, and those who co-operated well with the examiners were included in the study. Children who had previously undergone orthodontic or occlusal guidance treatment and those with a history of current or previous systemic diseases or craniofacial anomalies were excluded.

Among all children (*n* = 2,974), a remarkable proportion (*n* = 739) did not meet the inclusion criteria and were excluded. The remaining children (*n* = 2,235, 1,185 [53.02 %] boys and 1,050 [46.98 %] girls) were included in the research for further investigations. The mean age of the participants was 4.82 (SD, 1.76; range, 2.63–6.12) years.

### Ethics approval and consent to participate

The parents of the participating children were informed of the objectives of this research, and written informed consent was obtained from all the parents prior to conducting the study. The study was approved by the Ethical Committee of the School of Stomatology, Fourth Military Medical University, Republic of China (committee’s reference number: IRB-REV-2014068).

### Criteria of different types of malocclusion and associated variables

The clinical examinations for different types of malocclusion and occlusive variables such as fused teeth, insufficient abrasion of the primary canines, primate space, and caries in the primary teeth and premature exfoliation of primary teeth were performed by trained and calibrated clinicians using a mouth mirror, a graduated periodontal probe, and a tongue blade. The diagnosis criteria for each type of malocclusion were adopted from a previous study [20], as shown in Table 1.

**Table 1:** Types of malocclusion and related diagnostic criteria

	Types	Diagnosis criteria
Sagittal anomalies	Anterior crossbite	Recorded when the lower incisors or canines are in front of the corresponding upper teeth
	Edge-to-edge occlusion	Recorded when the maxillary incisors and the corresponding mandibular incisors are occluded in an edge-to-edge status, without overbite or overjet
	Increased overjet	Recorded when the distance from the most labial point of the incisal edge of the maxillary incisors to the most labial surface of the corresponding mandibular incisors is more than 3 mm, measured to the nearest 0.5 mm
Vertical anomalies	Open bite	Recorded when the upper and lower incisors are vertically separated regardless of the extent, without any overbite
	Deep overbite	Recorded when the vertical overlap of the incisors, when the posterior teeth are in contact is more than two thirds covered or when the overlap is more than 3 mm, measured to the nearest 0.5 mm
Transversal anomalies	Posterior crossbite	Recorded when the buccal cusps of the maxillary molars occlude lingually to the buccal cusps of the mandibular antagonists in at least one pair of teeth, uni- or bilaterally. Teeth in an edge-to-edge position were also included
	Midline deviation	Recorded when the position of the mandibular midline had a more than 2-mm discrepancy relative to the maxillary midline, with the posterior teeth in full occlusion

The diagnostic criteria for fused teeth were defined as two teeth with fused enamel or dentin with a forking crown. Insufficient abrasion of the primary canines was noted when the canine cusp was significantly higher than the normal occlusal plane, leading to an early occlusal contact with antagonistic teeth. Primate spaces were located mesial to the upper canines and distal to the lower canines. No visible primate space was considered as the absence of the primate space. In this study, both decayed and filled carious primary teeth were defined as carious [21]. Premature exfoliation of primary teeth was defined as loss of a tooth due to diseases or injuries outside the physiological exfoliation period.

### Design of questionnaire for parents

A questionnaire was designed focusing on the demographic characteristics of the children such as gender, birth place (urban/rural), parental education level (high school and below/undergraduate/master and above), monthly familial income, and parental attitude toward the problem of malocclusion (positive/not concerned). In addition, the feeding methods of the children (such as breast milk/milk powder/both), feeding postures (sitting/half sitting/flat/unfixed), pacifier use (yes/no), and delivery methods (such as natural labor/cesarean section) were investigated in this questionnaire. Moreover, the dental visit experiences of the children including the visiting frequency, the facility visited, and the visiting experience (pleasant/unpleasant) were assessed. Finally, the parents were asked regarding current or previous improper oral habits of their children, such as regular tongue thrusting, finger sucking, bruxism, mandibular prognathism, lip biting, and mouth breathing. The questionnaires were completed by the parents of the participating children by following the instructions and under the observation of trained examiners. Each of the improper habits was explained by the examiners and any queries raised by the participants were satisfactorily addressed.

### Data collection

Six examiners, who were all faculty members at the Department of Pediatric Dentistry, School of Stomatology, Fourth Military Medical University, Republic of China, participated in this study. Prior to the investigation, serial training sessions were arranged to clarify the purpose of this study, creating awareness about the correct methods for examining primary teeth, and guiding parents to fill the questionnaires accurately. In order to test the intra-examiner reproducibility, following four weeks of their initial examination, 30 children were selected for re-examination. The results showed a high intra-examiner agreement for each type of malocclusion (i.e., maximum and minimum Kappa values were 1.00 and 0.73, respectively). The inter-examiner reliability was determined by two observers who independently evaluated 30 children on the same day. All measurements showed a high degree of inter-examiner reliability, with the intraclass correlation coefficients higher than 0.88.

### Statistical analysis

Data were recorded and analyzed using the SPSS statistical software version 22.0. For different types of malocclusions, frequencies and percentages were generated; correlations between the selected variables and malocclusion were determined using the Chi-square test. Statistical significance was considered when *P* < 0.05. Statistically significant independent variables in the Chi-square test were further analyzed using binary logistic regression taking malocclusion as a dependent (*P* < 0.05), and odds ratios (ORs) were then obtained in addition to calculating 95 % confidence intervals (CIs).

## Results

### Prevalence of different types of malocclusion

Normal occlusion was observed in only 753 children (33.69 %), while 1,482 (66.31 %) children had one or more types of anomalies.

The general prevalence of malocclusion is presented in Table 2. The most common type of malocclusion in the sagittal direction was the increased overjet (34.99 %), while the most prevalent malocclusions in the vertical and transverse directions were deep overbite (37.58 %) and midline deviation (25.32 %), respectively. Deep overbite was the most common type of malocclusion among children with primary dentition. The prevalence of posterior crossbite, anterior crossbite, and anterior open bite was 7.56, 6.80 and 6.98 %, respectively. The prevalence of the anterior edge-to-edge occlusion was the lowest (2.46 %).

**Table 2:** Prevalence of different types of malocclusion

Type	N	Percentage (%)
Malocclusion in sagittal direction		
Anterior crossbite	152	6.80
Edge to edge occlusion	55	2.46
Increased overjet	782	34.99
Malocclusion in transversal direction		
Posterior crossbite	169	7.56
Midline deviation	566	25.32
Malocclusion in vertical direction		
Deep overbite	840	37.58
Open bite	156	6.98

### Relationship between different types of malocclusion and demographic characteristics

The prevalence of malocclusion among children with different demographic characteristics is shown in Table 3. It indicated that the prevalence in urban children was significantly lower than that in children residing in rural areas (61.59 % vs. 76.29 %). The highest prevalence (77.91 %, *P* < 0.001) was observed in the group of children with monthly familial income less than 2,000 yuan. No statistically significant (*P* > 0.05) association was observed between malocclusion and parental education level or gender.

**Table 3:** Prevalence of different types of malocclusion based on demographic characteristics

Variables	Prevalence of malocclusion (present/total)	*χ* ^2^	P	Prevalence of anterior crossbite (present/total)	Prevalence of edge to edge occlusion (present/total)	Prevalence of increased overjet (present/total)	Prevalence of posterior crossbite (present/total)	Prevalence of midline deviation (present/total)	Prevalence of deep overbite (present/total)	Prevalence of open bite (present/total)
Gender										
Male	65.49 (776/1185)			6.58 (78/1185)	2.36 (28/1185)	33.33 (395/1185)	7.26 (86/1185)	28.10 (333/1185)	42.45 (503/1185)	7.26 (86/1185)
Female	67.24 (706/1050)	0.766	0.382	7.05 (74/1050)	2.57 (27/1050)	36.86 (387/1050)	7.90 (83/1050)	22.19 (233/1050)	32.10 (337/1050)	6.67 (70/1185)
Birth Place										
Urban	61.59 (935/1518)			6.06 (92/1518)	1.84 (28/1518)	36.96 (561/1518)	5.47 (83/1518)	22.86 (347/1518)	39.26 (596/1518)	8.17 (124/1518)
Rural	76.29 (547/717)	47.078	<0.001	8.37 (60/717)	3.77 (27/717)	30.82 (221/717)	11.99 (86/717)	30.54 (219/717)	34.03 (244/717)	4.46 (32/717)
Parental education level										
High school and below	67.81 (394/581)			8.09 (47/581)	2.41 (14/581)	38.55 (224/581)	7.92 (46/581)	29.09 (169/581)	39.93 (232/581)	8.26 (48/581)
Undergraduate	64.95 (921/1418)			5.71 (81/1418)	2.26 (32/1418)	31.88 (452/1418)	6.63 (94/1418)	22.14 (314/1418)	34.56 (490/1418)	5.57 (79/1418)
Graduate and above	70.76 (167/236)	3.856	0.145	10.17 (24/236)	3.81 (9/236)	44.92 (106/236)	12.29 (29/236)	35.17 (83/236)	50.00 (118/236)	12.29 (29/236)
Familial monthly income (Yuan)										
< 2000	77.91 (261/335)			9.85 (33/335)	3.28 (11/335)	47.16 (158/335)	12.84 (43/335)	33.43 (112/335)	52.84 (177/335)	11.64 (39/335)
2000–5000	65.98 (516/782)			6.01 (47/782)	2.17 (17/782)	30.82 (241/782)	6.01 (47/782)	17.77 (139/782)	32.74 (256/782)	6.27 (49/782)
5000–8000	57.97 (389/671)			4.92 (33/671)	1.34 (9/671)	25.93 (174/671)	6.11 (41/671)	22.95 (154/671)	28.02 (188/671)	4.02 (27/671)
> 8000	70.69 (316/447)	44.937	<0.001	8.72 (39/447)	4.03 (18/447)	46.76 (209/447)	8.50 (38/447)	36.02 (161/447)	48.99 (219/447)	9.17 (41/447)

### Factors associated with oral examination and malocclusion

The results of Chi-square test indicated that children with insufficient abrasion of the primary canines, those lacking primate spaces, and those with caries in the primary teeth had a higher prevalence of malocclusion (*P* < 0.05, Table 4). No significant difference in malocclusion was detected between groups of children with premature exfoliation of primary teeth or fused teeth (*P* > 0.05, Table 4).

**Table 4:** Bivariate analysis of factors associated with oral examination, improper oral habits, feeding and delivery methods, dental visit experience, and malocclusion

	Variables	Prevalence of malocclusion (present/total)	*χ* ^2^	*P*
Oral examination	Fused tooth			
	Present	65.18 (73/112)		
	Absent	66.37 (1409/2123)	0.067	0.795
	Insufficient abrasion of primary canines			
	Present	73.28 (181/247)		
	Absent	65.44 (1301/1988)	6.040	0.014
	Primate space			
	Present	48.13 (775/987)		
	Absent	80.69 (707/1248)	117.996	<0.001
	Caries among primary teeth			
	Present	68.08 (1171/1720)		
	Absent	60.39 (311/515)	10.499	0.001
	Premature exfoliation of primary teeth			
	Present	66.36 (73/110)		
	Absent	66.31 (1409/2125)	0.00016	0.990
Improper oral habits	Tongue thrusting			
	Present	84.38 (54/64)		
	Absent	65.78 (1428/2171)	9.626	0.002
	Mouth breathing			
	Present	77.99 (163/209)		
	Absent	66.09 (1319/2026)	14.083	<0.001
	Bruxism			
	Present	64.76 (441/681)		
	Absent	66.99 (1041/1554)	1.055	0.304
	Mandibular prognathism			
	Present	82.86 (29/35)		
	Absent	66.05 (1453/2200)	4.359	0.037
	Lip biting			
	Present	74.04 (77/104)		
	Absent	65.93 (1405/2131)	2.917	0.088
	Finger sucking			
	Present	54.25 (287/400)		
	Absent	48.50 (1195/2835)	6.457	0.011
Feeding and delivery methods	Feeding methods			
	Breast milk	23.10 (131/567)		
	Milk powder	76.78 (443/577)		
	Both	83.23 (908/1091)	641.829	<0.001
	Feeding postures			
	Sitting	66.86 (577/863)		
	Half sitting	64.10 (557/869)		
	Flat	69.51 (285/410)		
	Unfixed	67.74 (63/93)	3.990	0.263
	Use of pacifier			
	Yes	73.98 (307/415)		
	No	64.56 (1175/1820)	13.410	<0.001
	Delivery methods			
	Natural labor	62.97 (670/1064)		
	Caesarean	69.34 (812/1171)	10.133	0.001
Dental visiting experience	Frequency of visiting dental clinic			
	Never	80.28 (635/791)		
	Only when needed	62.71 (676/1078)		
	3 months	38.10 (16/42)		
	Half a year	47.24 (137/290)		
	Irregular	52.94 (18/34)	140.227	<0.001
	Facility of the visited dental clinic			
	Public hospital	59.10 (789/1335)		
	Community hospital	57.83 (48/83)		
	Private practice	38.46 (10/26)	4.505	0.105
	Had unpleasant dental visit experience			
	Yes	74.76 (154/206)		
	No	55.98 (693/1238)	25.685	<0.001
	Parental attitude toward the problem of malocclusion			
	Pay positive attention	52.81 (293/409)		
	Not concerned	69.33 (1189/1826)	6.365	0.012

### Relationship between improper oral habits and malocclusion

The relationship between improper oral habits and malocclusion was also confirmed by Chi-square test (Table 4). The results showed that children with tongue thrusting and mandibular prognathism had a higher probability of malocclusion (*P* < 0.05) compared to normal children. Moreover, mouth breathing or finger sucking were associated with a higher prevalence of malocclusion in the primary dentition (*P* < 0.05). The data showed that bruxism together with lip biting did not display any statistical significance with malocclusion (*P* > 0.05).

### Relationship between feeding/delivery methods and malocclusion

With regard to the feeding methods (Table 4), Chi-square test demonstrated that the majority of the children were fed on both breast milk and milk powder during infancy. The prevalence of malocclusion showed a significant difference between these groups (*P* < 0.001), while breastfed children had the lowest incidence of malocclusion (23.10 %). With regard to the feeding postures, majority of the children were fed in sitting or half-sitting positions compared to flat or unfixed postures. However, there was no statistically significant difference among these groups (*P* > 0.05). Most children with primary dentition had never used a pacifier. However, the prevalence of malocclusion was higher than in those who did not use pacifiers as infants (73.98 % vs. 64.56 %, *P* < 0.001). With regard to the delivery method, children born via natural labor were less likely to experience malocclusion in the primary dentition than children born via cesarean section (*P* < 0.05).

### Relationship between dental visit experience and malocclusion

The experience of previous dental visits was also investigated for its association with malocclusion (Table 4). With regard to the visiting frequency, children who never underwent a routine oral examination or never visited dental clinics when necessary had a significantly higher rate of malocclusion (*P* < 0.05) compared to children who visited dental clinics more frequently at regular intervals of three to six months. In terms of the experience of visiting dental clinics, 14.27 % of the children reported unpleasant experiences. Surprisingly, children in this group had a higher prevalence of malocclusion (*P* < 0.05). Majority of the parents (92.45 %) preferred public hospitals for their children, followed by community hospitals (5.75 %) and private practices (1.80 %). The hospital type (public, community, or private) had no remarkable effects on the prevalence of malocclusion among children with primary dentition (*P* > 0.05). Regardless of the education level of the parents and the monthly familial income, proper parental monitoring and understanding of malocclusion were correlated with a lower prevalence of malocclusion in children compared to children whose parents who lacked these behaviors (*P* < 0.05).

### Binary logistic regression analysis of associated factors and malocclusion

The results of conditional binary logistic regression analysis (Table 5) indicated that rural children had a 1.741 times higher risk of suffering from malocclusion than urban children (*P* < 0.05, Table 5). There was no significant difference in the prevalence of malocclusion based on the familial economic status (*P* > 0.05). Insufficient abrasion of the primary canines and caries played a critical role in the morbidity of malocclusion (*P* < 0.05, Table 5). Compared to normal children, the risk of malocclusion among those who suffered from insufficient abrasion of the primary canines or caries was higher (OR = 1.465 and 2.045, respectively). Although the prevalence of malocclusion was higher in the group of children who had lost their primate space, further logistic regression analysis signified no statistical significance. Results of improper oral habits confirmed that children with tongue thrusting and mandibular prognathism had OR = 2.833 and 2.621, respectively. Moreover, children with a habit of finger sucking were at a higher risk (1.573 times) of malocclusion (*P* < 0.05, Table 5). The data showed that mouth breathing did not show any statistical significance with malocclusion in primary dentition. Breast feeding and natural labor did have a positive preventive effect toward malocclusion (*P* < 0.05, Table 5). Although children who had used pacifiers showed an incidence of malocclusion, it was not statistically significant (*P* > 0.05). Regression analysis further confirmed that proper parental monitoring and understanding of malocclusion were correlated with its lower prevalence than in children of parents who lacked these behaviors (Table 5, *P* < 0.05).

**Table 5:** Binary logistic regression analysis of relative factors and malocclusion

Variables	Prevalence of malocclusion (present/total)	β	S.E.	P	Odds ratio (95 % CI)
Birth place					
Urban	61.59 (935/1518)				
Rural	76.29 (547/717)	1.825	0.309	0.027	1.741 (1.384–2.162)
Insufficient abrasion of primary canines					
Present	73.28 (181/247)				
Absent	65.44 (1301/1988)	0.363	0.395	0.037	1.465 (1.153–.894)
Caries among primary teeth					
Present	68.08 (1171/1720)				
Absent	60.39 (311/515)	1.296	0.78	<0.001	2.045 (1.665–2.539)
Tongue thrusting					
Present	84.38 (54/64)				
Absent	65.78 (1428/2171)	0.725	0.114	0.041	2.833 (1.640–3.649)
Mandibular prognathism					
Present	82.86 (29/35)				
Absent	66.05 (1453/2200)	0.855	0.101	0.023	2.621 (1.574–3.689)
Finger sucking					
Present	54.25 (287/400)				
Absent	48.50 (1195/2835)	0.642	0.173	<0.001	1.573 (1.098–2.014)
Feeding methods					
Breast milk	23.10 (131/567)				
Milk powder	76.78 (443/577)				
Both	83.23 (908/1091)	1.090	0.347	0.012	3.614 (3.087–4.596)
Delivery methods					
Natural labor	62.97 (670/1064)				
Caesarean	69.34 (812/1171)	0.596	0.047	0.028	1.847 (1.323–2.451)
Parental attitude toward the problem of malocclusion					
Paid positive attention	52.81 (293/409)				
Not concerned	69.33 (1189/1826)	1.080	0.452	0.039	1.572 (1.201–2.512)

## Discussion

This epidemiological study revealed a high prevalence of malocclusion in children with primary dentition (66.31 %) residing in Xi’an city. Further statistical analysis revealed that factors such as insufficient abrasion of the primary canines and caries in the primary teeth were associated with malocclusion among these children. In addition, improper oral habits such as tongue thrusting, mandible prognathism, and finger sucking contributed to malocclusion. Children born by natural labor and breastfed children had a lower prevalence of malocclusion. A positive attitude of parents toward this problem is likely to reduce the prevalence of malocclusion in the primary dentition.

In the primary dentition, the occlusive relationship is influenced by a dynamic set of forming and adjusting forces [22]. The incidence of suffering from one or more types of malocclusion is high during this period, as demonstrated in our study (66.31 %) and previous studies [23, 24]. The prevalence in the current study was much higher than that in the Brazilian population (46.2 %) [23]. The differences between our study and other published articles [18, 19, 23, 24] could be attributed to the use of variable criteria for the classification of malocclusion; however, more attention needs to be paid to the overall problem.

Deep overbite and increased overjet had the highest morbidity among our subjects; however, the cephalocaudal gradient of craniofacial development should also be taken into consideration. In the preliminary stage of occlusal establishment, the lower dental arch is located in a distal position, exhibiting a vertical or distal terminal plane for the primary molars [25], corresponding to Angle’s II occlusion for the permanent dentition. Children with primary dentition usually have a higher risk of developing increased overjet. Insufficient distance between the dental arches may result in a deep overbite [26]. With the development of roots and forward movement of the lower dentition, the occlusion noted as deep overbite and increased overjet can gradually normalize. Borzabadi-Farahani et al. [5] performed a similar investigation on malocclusion in permanent dentition and reported a similar prevalence of different types of malocclusion; however, the prevalence of increased overjet and class II division I malocclusion in primary dentition was lower than the prevalence of increased overjet reported by our study. As previously mentioned, change in occlusion in the primary dentition is a dynamic process. Therefore, dynamic observations are also needed to differentiate between temporary malocclusion and pathological malocclusion in this period.

With regard to parental characteristics, our study showed that education level had no relationship with malocclusion. Regardless of the education level, positive parental attitude toward oral health is important. Monitoring of primary dentition in a regular and timely manner was associated with a lower incidence of malocclusion. This finding was in accordance with the traditional perception that positive parental participation would lead to early prevention and timely treatment of malocclusion. A higher prevalence of malocclusion was observed among children born and living in the rural areas who were less likely to visit pediatric dentists at regular intervals. Our results are in agreement with Guan et al. study, which reported an increased prevalence of dental caries associated with lower socioeconomic classes but not with parental education levels [27].

Children having primary dentition with insufficient abrasion of the primary canines and a high caries risk showed a higher incidence of malocclusion. Our finding that children who suffered from caries had a higher incidence of malocclusion was slightly different from those of previous studies [28, 29]. Feldens et al. [28] confirmed that there was a correlation between caries and handicapping malocclusion, and between maxillary irregularity and abnormal molar relationship in subjects with permanent dentition. However, no statistically significant correlation was observed in malocclusion in the primary dentition. Singh et al. [29] reported similar findings, that only those who had caries in the mixed and young permanent dentition had a higher probability of suffering from malocclusion. Borzabadi-Farahani et al. [30] found a different interrelationship among children in the older age group, but not among those in lower socioeconomic status groups. In contrast, our study reported that socioeconomic status was not an associated factor for malocclusion. Although we considered a different evaluating indicator for socioeconomic status, the relationship between caries experience and malocclusion should be assessed in a wider context of socioeconomic status and background factors. The association of dental caries with malocclusion is controversial. On the one hand, the authors of this study believed that caries would lead to changes in the length or width of the dentition, thereby leading to abnormal alignment of the primary teeth. On the other hand, caries in the primary molars might lead to crown disintegration, subsequently changing the normal chewing habits of children in their primary dentition, which, to a certain degree, could alter the position of the temporomandibular joint, ultimately resulting in malocclusion.

Primary anterior teeth, especially the primary canines, typically have insufficient abrasion that may affect the relationship among the anterior teeth. Consequently, the mandible may not move back to a relatively normal position, leading to a crossbite of the anterior primary teeth. Caries in the primary dentition, especially primary molars, is interrelated to insufficient abrasion of the primary canines. Soft food items consumed by children may contribute to the insufficient abrasion of the primary canines along with caries. Additionally, severe caries could decrease the intermaxillary height, leading to the primary canines being positioned relatively higher corresponding to insufficient abrasion.

Several studies [31–33] have reported a higher incidence of malocclusion among children with improper oral habits compared to normal children. Some of the most common improper oral habits, such as lip biting, finger sucking, and tongue thrusting have been correlated with various types of malocclusions including inclined teeth, dental arch constriction, open bite, and increased interdentium [34]. However, the current study showed slightly different results; it was observed that in children with primary dentition, malocclusion had no statistical significant correlation with bruxism and lip biting. Our results were supported by Hella et al. [35], who first stated that most lip habits would not lead to an occlusion problem unless they attained a certain degree of strength, frequency, and duration. Hence, if lip habits lead to malocclusion, their effects would appear only in the permanent or mixed dentition.

From the parental questionnaires, it was noted that breastfed children in their primary dentition were less likely to suffer from malocclusion. These findings are similar to another Puerto Rican study [36], which reported that an increased duration of breastfeeding was associated with a decline in the proportion of children suffering from malocclusion. Some scholars [37] believed that compared to other feeding patterns, breastfeeding could significantly reduce the risk of caries. Considering our previous finding that caries may be related to the development of malocclusion, breastfeeding could have a significantly positive effect on malocclusion in the primary dentition.

The World Health Organization (WHO) reported that the overall rate of cesarean births in China increased to 46.2 % from October 2007 to May 2008, ranking the country highest in the world for cesarean delivery rate. In some cities, this proportion is as high as 70 % [38], which is significantly higher than the 15 % threshold set by the WHO. Our results showed that natural labor may be associated with a lower incidence of malocclusion in the primary dentition. Previously, Cattaneo et al. [39] investigated 106 adults to further explore the relationship between malocclusion and delivery methods. The results showed that none of the subjects with malocclusion were delivered naturally. This could be attributed to the maxillofacial trauma during non-natural deliveries. Although further studies are warranted, data from this study and those of other articles suggested that natural birth and breastfeeding may reduce the prevalence of malocclusion.

This study had several limitations. Firstly, there was more than one type of malocclusion in the primary dentition (Table 2). Individual variations can further complicate the investigation of various malocclusions. However, in this study, it was not explored whether one factor was associated with a certain type of malocclusion. Thus, the results could only partly illustrate the influencing relationships. Secondly, although the examiners did provide explanations when parents were investigated about the improper oral habits of their children, the results could still be subjective to some extent. In addition, some of the parents might not have been able to distinguish between habits and occasional behavior. In other words, the duration and frequency of improper oral habits were not taken into consideration in this study. Therefore, further analysis of the data might be required. Finally, few of the primary dentition malocclusions such as crowding (one of the most commonly seen malocclusion) among children in older age group [5, 40] were not included in this study; therefore, further investigation in this regard is also warranted.

Compared to permanent dentition, fewer studies have focused on malocclusion in the primary dentition. In addition, most of these studies cannot represent the general prevalence of a particular region because of small sample sizes or investigation indices. In this study, the stratified cluster sampling method was used. Moreover, a suitable sample size representative of the general prevalence in the city of Xi’an was ensured. Besides, as Angle’s malocclusion classification uses the molar relationship for permanent dentition, it does not incorporate vertical and transverse abnormalities [41]. Thus, Bjoerk’s classification [20] better suits the primary dentition.

## Conclusions

The prevalence of malocclusion among pre-school children in Xi’an is higher than that in pre-school children in other geographical parts of China. Therefore, early attention to the development of occlusion and necessary interventions toward its influencing factors are important to reduce its prevalence and further adverse effects. This study provided considerable information regarding the prevalence of malocclusion and its contributing factors in the primary dentition. These theoretical evidences could be taken into account for future studies focused on preventing malocclusion in children.

## Abbreviations

CI: Confidence interval
ICC: Intraclass correlation coefficient
OR: Odds ratio
WHO: World Health Organization
